# Effect of parental supervision of infants at age 4 to 6 months on injuries at age 4 to 12 months

**DOI:** 10.1038/s41598-022-14321-8

**Published:** 2022-06-17

**Authors:** Won Seok Lee, Kyung Suk Lee, Eun Kyo Ha, Ju Hee Kim, So Min Shim, Seung Won Lee, Man Yong Han

**Affiliations:** 1 Department of Pediatrics, CHA Ilsan Medical Center, CHA University, Goyang, Republic of Korea; 2 Department of Data Science, Sejong University College of Software Convergence, 209, Neungdong-ro, Gwangjin-gu, Seoul, 05006 Republic of Korea; 3 Department of Pediatrics, Hanyang University Guri Hospital, Hanyang University College of Medicine, Guri, Republic of Korea; 4 Department of Pediatrics, Hallym University Kangnam Sacred Heart Hospital, Seoul, Republic of Korea; 5 Department of Pediatrics, Kangdong Sacred Heart Hospital, Hallym University College of Medicine, Seoul, Korea; 6 Department of Pediatrics, School of Medicine, CHA Bundang Medical Center, CHA University, 59, Yatap-ro, Bundang-gu, Seongnam-si, Gyeonggi-do 13496 Republic of Korea; 7 Department of Precision Medicine, Sungkyunkwan University School of Medicine, Suwon, Republic of Korea

**Keywords:** Medical research, Risk factors

## Abstract

This study analyzed the effect of parental supervision of infants at age 4 to 6 months on injuries at age 4 to 12 months. Among all Korean children born during 2008–2009, 464,326 (50.6%) infant had parents who responded to a questionnaire that surveyed their safety and supervision when infant were 4 to 6 months-old. Based on questionnaire score, infant were divided into “safe” or “unsafe” group. 1:1 propensity score matching was used to balance the groups, and injury diagnosis and treatments were analyzed. After matching, we examined the records of 405,862 infant. The unsafe group had significantly increased risk ratios (RRs) for injury of head/neck (RR: 1.06), trunk/abdominopelvic region (RR: 1.12), upper extremities (RR: 1.04), and from burn and frostbite (RR: 1.10). The risks of a wound and fracture and foreign body injury were significantly greater in infant whose parents sometimes left them alone (RR: 1.15 and 1.06, respectively), and whose parents did not always keep their eyes on them (RR: 1.04 and 1.13, respectively). Infant whose parents had a hot drink when carrying them had an increased risk of burn injuries (RR: 1.21). Injuries were less common in infant whose parents provided more supervision.

## Introduction

Injuries are the main reasons children are referred to emergency departments and the most common cause of disability and death during childhood^1^. Injury and violence are responsible for about 950,000 deaths per year worldwide in individuals younger than 18 years, and unintentional injuries account for almost 90% of these cases^2^. Analysis of children younger than 15 years-old indicated that children under 12 months-old have the highest injury rate^3^ and the poorest injury outcomes, including mortality^4^.

Children of different ages are in different developmental stages, and this must be considered when assessing parental awareness of child safety. For example, infants (< 12 months-old) are generally not capable of making decisions on their own that prevent injuries, so the adult caregivers are responsible for keeping them safe. A study of Organization for Economic Cooperation and Development (OECD) countries reported that the majority of fatal injuries in infants occurred at home^5^, and that most of these could have been prevented if caregivers had taken greater precautions^6^. Parents can play a key role in reducing the likelihood of injuries at home by close supervision of their children’s activities^7^. The role of parents in assuring the safety and reducing the risk of injury in their children depends on the age and development stage of the children, the occupation and socioeconomic status of the parents, and the specific cultural environment^8^. The risk of child injury is influenced by parental awareness, attitudes, and behaviors related to the safety of their children^8–10^. A meta-analysis reported that increased education or intervention by parents effectively prevented childhood injuries and improved safety at the home^11^.

The aim of this study was to use safety-related questionnaires that were administered to Korean parents and nationwide population-based data to evaluate the effect of parental supervision of children and the need for injury-related treatments when the children were 4 to 6 months-old on injuries and injury-related treatments of these children when they were 4 to 12 months-old.

## Methods

### Study design and data sources

Among all children Korean born from 2008 to 2009, infants were included if their parents participated in the safety-related questionnaire in the National Health Screening Program for Infants and Children (NHSPIC) when they were 4 to 6 months-old. The NHSPIC is a screening program designed to assess and provide education regarding the health, nutrition, development, and safety of Korean children^12^. The medical records of all subjects were collected from the Korean National Health Insurance Service (NHIS). These data included demographic and clinical characteristics and diagnostic and treatment information related to childhood injuries using codes from the *International Classification of Diseases 10th revision* (ICD-10). The answers to the safety-related questionnaire (provided by parents or caregivers) and other results were checked by clinical physicians who were registered in medical institutions.

The Institutional Review Board (IRB) of CHA University Bundang CHA Hospital (IRB number 2018-10-005) approved the protocol of this study, and the waiver of informed consent from participants, as all data in the Korean National Health Insurance Service (NHIS) and National Health Screening Program for Infants and Children (NHSPIC) were anonymized. The de-identified individual data were used only for research purposes, and this research was conducted with ethical clearance under the current National Health Insurance Act^13^. This retrospective study was performed in accordance with all relevant guidelines and regulations.

### Study population

**Figure 1:**
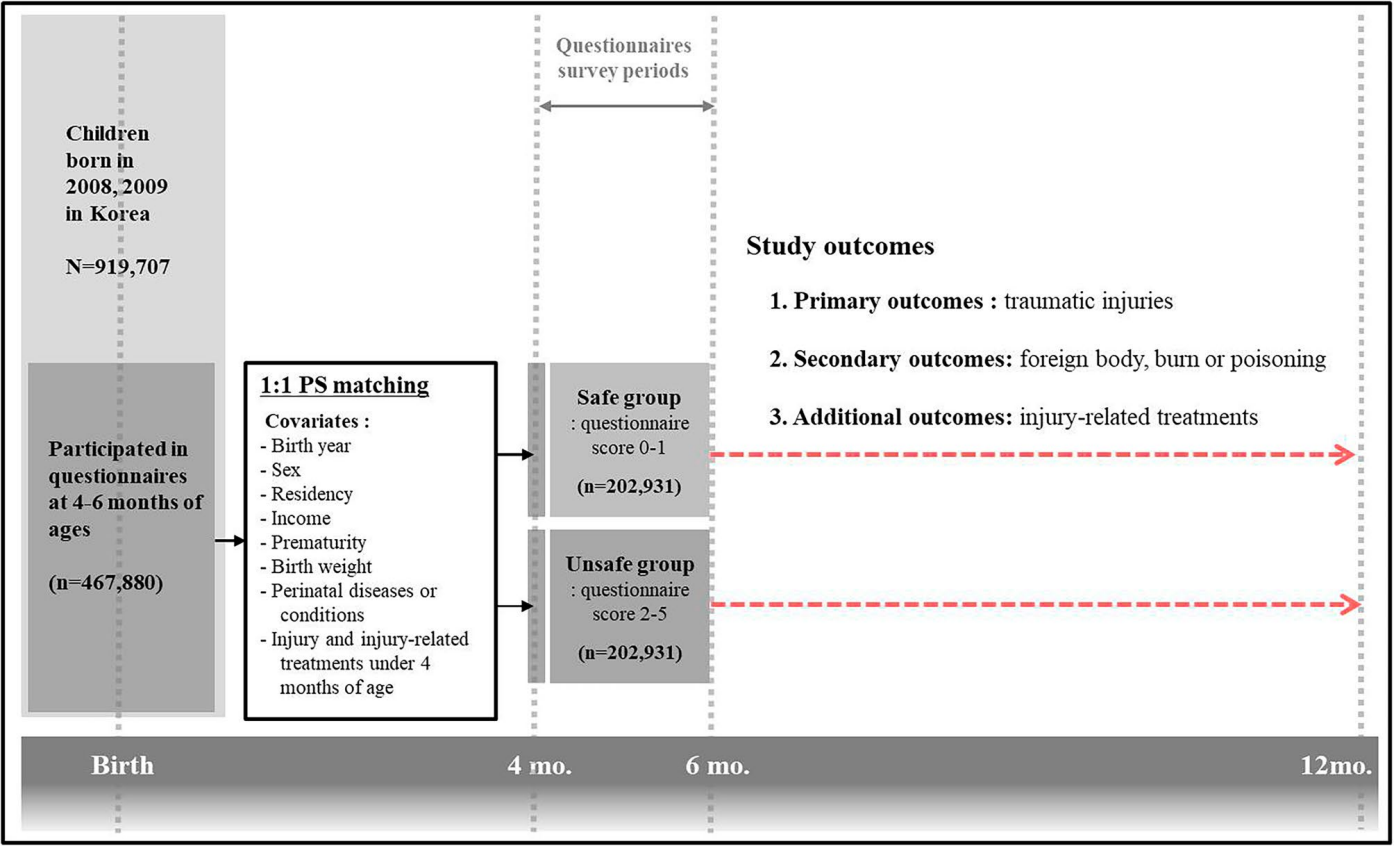
Study design. A total of 917,707 children were born in Korea from 2008 to 2009, and the parents or caregivers of 467,880 of them answered a safety-related questionnaire when they were 4 to 6 months-old. Propensity score-matching was used to balance the covariates between the “safe group” and the “unsafe group”. These covariates included demographic and clinical characteristics, injury diagnosis, and treatment for an injury before age 4 months (Supplementary Table S1). After matching, 202,931 children were assigned to each group. The primary outcome was traumatic injury, the secondary outcome was non-traumatic injury, and the additional outcome was treatment for an injury.

**Figure 2:**
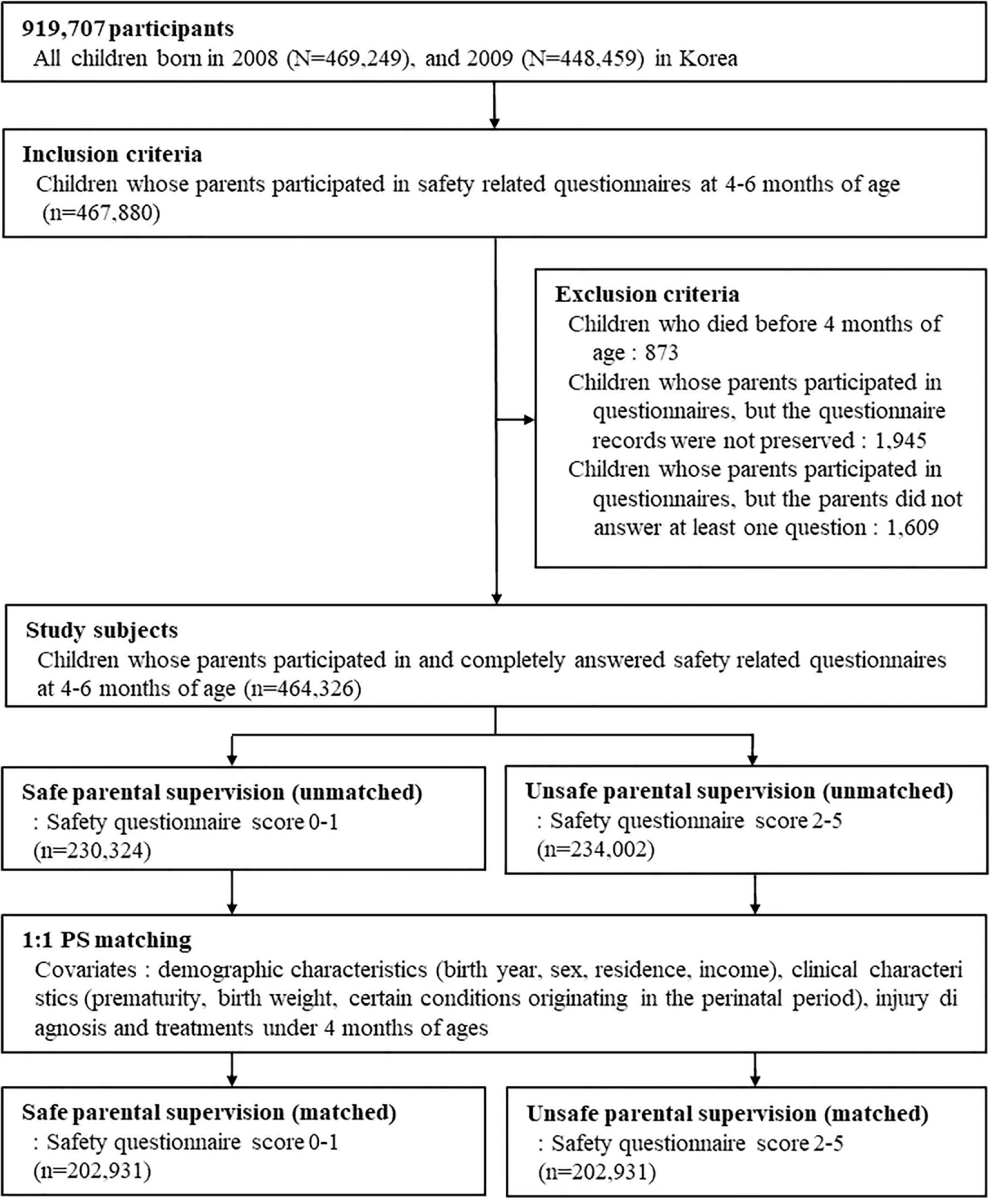
Disposition of participating children.

A total of 917,707 children were born in Korea from 2008 to 2009. The 467,880 infants whose parents or caregivers answered the safety-related questionnaire when they were 4 to 6 months-old were initially included. Children were excluded if they died within 4 months of age (n = 873), if the questionnaire records were lost (n = 1945), or if the parents did not answer one or more questions (n = 1609). Finally, 464,326 infants who were 4 to 6 months-old met the inclusion and exclusion criteria (Figs. 1, 2).

### Safety questionnaire

There were 5 questions that asked about childhood injuries and safety during the screening program when children were 4 to 6 months-old. Each question had two possible answers; a score of “0” indicated “safe” and a score of “1” indicated “unsafe”. These questions were as follows (Supplementary Table S1): Do you always use a car seat when your child rides in a car (yes: 0, no: 1)? Do you use a baby walker (no: 0, yes: 1)? Have you ever left your child alone on the bed or couch for a while (no: 0, yes: 1)? Do you keep an eye on your child all the time, including while playing and sleeping (yes: 0, no: 1)? Have you ever held a hot drink when carrying your child (no: 0, yes: 1).

The subjects were divided into two groups according to the total questionnaire score. There were 230,324 infants who scored 0 to 1 (safe group), and 234,002 infants who scored 2 to 5 (unsafe group). After 1:1 propensity score matching, 202,931 children were assigned to each group (Figs. 1, 2).

### Primary, secondary, additional outcomes, and subgroup analysis

The primary outcome was a total of traumatic or non-traumatic injury at the age of 4 to 12 months. The secondary outcomes were individual traumatic and non-traumatic injuries. The diagnosis of traumatic injuries considered four parts of body: head and neck (ICD code S00–S19), trunk and abdominopelvis (ICD code S20–S39), upper extremities (ICD code S40–S69), and hip and lower extremities (ICD code S70–S99). Thus, these ICD-10 injury codes ranged from S00 to S99. The diagnosis of non-traumatic injuries consisted as followings: foreign body (ICD code T15–T19), burn and frostbite (ICD code T20–T35), and poisoning and toxic effects by drugs and biological substances, and unspecified effects of external causes (ICD code T36–78).

An additional outcome measure was treatment for an injury at a medical institution at the age of 4 to 12 months. The four analyzed treatments were: anesthesia, cast or splint, blood product transfusion, and ventilator support (Supplementary Appendix 1).

Subgroup analysis was conducted to identify the risk ratio (RR) of childhood injury according to the answer to each of the five safety-related questions. The four causes of injuries were: wound and fracture, foreign body, burn and frostbite, and poisoning. The RR of childhood injury was subdivided by ICD-10 codes of S00 to S99 and T15 to T78 (Supplementary Appendix 2).

### Covariates

The covariates included multiple demographic and clinical characteristics, type of injury before the age of 4 months, and type of treatment received for injury before the age of 4 months (Supplementary Table S2). Income quintile was determined by the amount of insurance co-payment, and ranged from 1 to 5 (highest). Residence at birth was classified as Seoul, a metropolitan area (Busan, Daegu, Incheon, Gwangju, Daejeon, and Ulsan), an urban area (area belonging to or related to a town or city), or a rural area (area far from large towns or cities). Clinical characteristics included prematurity (gestational period < 37 weeks), birth weight (kg, mean ± standard deviation), and conditions originating during the perinatal period (birth trauma, respiratory and cardiovascular disease, congenital malformations, and chromosomal abnormalities), based on ICD-10 code. The injury diagnosis (head and neck injury, upper and lower extremity injury, burn or frostbite injury, foreign body and poisoning injury), and the need for treatment (anesthesia, cast or splint, transfusion, or ventilator support) were also included as covariates.

### Statistical analysis

Propensity score matching was used to reduce potential confounders and to balance the baseline covariates of the “safe” and “unsafe” groups using a multivariable logistic regression. A sensitivity analysis was performed by adding the missing data, and this data included the information of following subjects: ‘children whose parents participated in questionnaires, but the questionnaire records were not preserved’ (n = 1945), and ‘children whose parents participated in questionnaires, but the parents did not answer at least on question’ (n = 1609). Between-group differences in baseline characteristics were compared using standardized differences in the matched and unmatched samples, and a difference greater than 10% was considered meaningful^14^. The risk ratios and 95% confidence intervals (CIs) were obtained using modified Poisson regression, and risk differences and 95% CIs were obtained using a binomial regression model with a log-link function. All statistical analyses were conducted using SAS software, version 9.4 (SAS Institute Inc.).

## Results

### Baseline characteristics of study subjects

We first compared the demographic and clinical characteristics of infants in the “safe” and “unsafe” groups (Table 1). After matching, all standardized differences were less than 10%. Then, we examined the diagnoses and treatments of children who were less than 4 months-old in the unmatched and matched groups (Supplementary Table S3). Among all 464,326 infants, 8482 (1.8%) had injuries before the age of 4 months, and the most common type was head injury (3040, 1.3%). Before and after matching, all standardized differences were less than 10%. Thus, the two groups in our matched analysis had no major imbalances in all examined demographic and clinical characteristics, and in injuries before the age of 4 months.

**Table 1:** Demographic and clinical characteristics of children in the main cohort.

	Unmatched data (N = 464,326)			Matched data (N = 405,862)^a^		
	Safe group N (%)^b^, (N = 230,324)	Unsafe group N (%)^b^, (N = 234,002)	Standardized difference, %^c^	Safe group N (%)^b^, (N = 202,931)	Unsafe group N (%)^b^, (N = 202,931)	Standardized difference, % ^c^
**Demographic characteristics**						
Birth year			**10.290**			1.400
2008	106,801 (46.4%)	120,651 (51.6%)		99,591 (49.1%)	101,009 (49.8%)	
2009	123,523 (53.6%)	113,351 (48.4%)		103,340 (50.9%)	101,922 (50.2%)	
Sex			1.380			− 0.139
Boy	118,425 (51.4%)	121,921 (52.1%)		105,077 (51.8%)	104,936 (51.7%)	
Girl	111,899 (48.6%)	112,081(47.9%)		97,854 (48.2%)	97,995 (48.3%)	
Residence at birth^d^			7.530			− 2.236
Seoul	61,990 (26.9%)	54,016 (23.1%)		52,491 (25.9%)	52,479 (25.9%)	
Metropolitan	51,840 (22.50%)	55,854 (23.9%)		47,144 (23.2%)	49,820 (24.6%)	
Urban	89,222 (38.7%)	93,837 (40.1%)		80,399 (29.6%)	79,546 (39.2%)	
Rural	25,457 (11.1%)	27,921 (11.9%)		22,897 (11.3%)	21,086 (10.4%)	
Income quantile^e^			** − 16.289**			− 1.231
1 (lowest)	15,574 (6.8%)	19,911 (8.5%)		15,119 (7.5%)	14,960 (7.4%)	
2	29,312 (12.7%)	37,712 (16.1%)		28,790 (14.2%)	30,149 (14.9%)	
3	58,096 (25.2%)	64,916 (27.7%)		56,574 (27.9%)	57,272 (28.2%)	
4	76,956 (33.4%)	70,376 (30.1%)		69,308 (34.2%)	67,345 (33.2%)	
5 (highest)	42,143 (18.3%)	33,532 (14.3%)		33,140 (16.3%)	33,205 (16.4%)	
**Clinical characteristics**						
Prematurity	13,679 (5.9%)	13,033 (5.6%)	1.617	11,205 (5.5%)	11,992 (5.9%)	− 1.667
Birth weight, kg (SD)^e^	3.20 (0.44)	3.21 (0.44)	2.500	3.20 (0.44)	3.20 (0.40)	0
**Condition originating during the perinatal period**^**f**^						
Maternal factors	6243 (2.7%)	6057 (2.6%)	0.721	5211 (2.6%)	5654 (2.8%)	− 1.356
Disorders related to length of gestation and fetus size	8658 (3.8%)	7845 (3.4%)	2.138	6756 (3.3%)	7307 (3.6%)	− 1.466
Birth trauma	2259 (1.0%)	2263 (1.0%)	0.087	1930 (1.0%)	2048 (1.0%)	− 0.592
Respiratory and cardiovascular disorders	14,626 (6.4%)	14,012 (6.0%)	1.492	12,180 (6.0%)	13,104 (6.5%)	− 1.889
Infection	35,674 (15.5%)	35,841 (15.3%)	0.349	31,019 (15.3%)	33,215 (16.4%)	− 2.997
Hemorrhagic and hematological disorder	78,800 (34.2%)	78,575 (33.6%)	1.281	68,871 (33.9%)	69,979 (34.5%)	− 1.153
Endocrine and metabolic disorder	11,225 (4.9%)	10,265 (4.4%)	2.222	9035 (4.5%)	9524 (4.7%)	− 1.145
Digestive disorder	7079 (3.1%)	7353 (3.1%)	− 0.410	6275 (3.1%)	6690 (3.3%)	− 1.177
Integument and temperature regulation problem	8591 (3.7%)	8687 (3.7%)	0.145	7506 (3.7%)	7983 (3.9%)	− 1.243
Congenital malformations or deformation	15,379 (6.7%)	14,783 (6.3%)	1.388	12,984 (6.4%)	13,803 (6.8%)	− 1.636
Chromosomal abnormality	25,497 (11.1%)	24,410 (10.4%)	2.154	21,494 (10.6%)	23,038 (11.4%)	− 2.456

*N* number, *SD* standard deviation.

Unless otherwise specified, baseline characteristics were assessed on the date of birth.

^a^Matched using inverse probability of exposure matching, based on propensity scores. The propensity score was estimated using multivariable logistic regression with covariates chosen a priori (Table S2).

^b^Values are reported as N (%) unless otherwise indicated.

^c^The difference between the groups divided by the pooled standard deviation; a value greater than 10% was considered meaningful.

^d^Obtained from the First National Health Screening Program of Infants and Children at age 4 to 6 months.

^e^Average income quintile of the neighborhood at birth.

^f^Conditions that originated during the perinatal period were identified by ICD-10 codes (Supplementary Appendix 3).

Significant values are in bold.

### Primary outcome: diagnosis of total injury (trauma or non-trauma)

We analyzed the diagnosis of all traumatic and non-traumatic injuries in children from the age of 4 to 12 months. In the unmatched data, 79,309 of 464,326 infants (17.1%) had injuries. There were significant differences between the safe and unsafe groups in the diagnosis of total injuries in the matched data (16.6% *vs.* 17.5%, p < 0.001) and in the unmatched data (16.6 vs. 17.5%, p < 0.001) (Table 2).

**Table 2:** Risk ratios for injuries and treatments in children aged 4 to 12 months.

	Unmatched data (N = 464,326)			Matched data (N = 405,862)^b^			
	Total events, N (%)	Safe group (N = 230,324), N (%)^d^	Unsafe group (N = 234,002), N (%)^d^	Safe group (N = 202,931) (%)^d^	Unsafe group (N = 202,931) (%)^d^	RR (mod Poisson)	
						Estimate	Wald 95% CI
**Diagnosis of total injury (trauma or non-trauma) in children aged 4 to 12 months (primary outcome)**^**a**^							
Total injury (trauma or non-trauma)	79,309 (17.1%)	**38,244 (16.6%)**^**†**^	**41,065 (17.5%)**^**†**^	**33,686 (16.6%)**^**†**^	**35,540 (17.5%)**^**†**^	**1.06**	**1.04 to 1.07**

	Unmatched data (N = 464,326)			Matched data (N = 405,862)^b^			
	Total events, N (cases/1000 person-months; 95% CI)	Safe group (N = 230,324), (cases/1000 person-months; 95% CI)^d^	Unsafe group (N = 234,002), (cases/1000 person-months; 95% CI)^d^	Safe group (N = 202,931), N (%)^d^	Unsafe group (N = 202,931), N (%)^d^	RR (mod Poisson)	
						Estimate	Wald 95% CI
**Risk ratios for traumatic injuries of different body legions in children aged 4 to 12 months (secondary outcome)**^**a**^							
Head and neck injury	36,205 (8.66; 8.57, 8.75)	**17,463 (8.39; 8.26, 8.53)**^**†**^	**18,742 (8.92; 8.78, 9.06)**^**†**^	**15,332 (7.56%)**^**†**^	**16,294 (8.03)**^**†**^	**1.06**	**1.04 to 1.09**
Trunk and abdominopelvic injury	3072 (0.74; 0.71, 0.76)	1434 (0.69; 0.65, 0.73)*	1638 (0.77; 0.73, 0.81)*	1261 (0.62%)*	1410 (0.69%)*	**1.12**	**1.04 to 1.21**
Upper extremity injury	16,182 (3.87; 3.81, 3.93)	7892 (3.80; 3.47, 3.64)*	8290 (3.94; 3.85, 4.03)*	6940 (3.42%)*	7192 (3.54%)*	**1.04**	**1.00 to 1.07**
Hip and lower extremity injury	421 (1.13; 1.10, 1.16)	2376 (1.13; 1.08, 1.18)	2345 (1.12; 1.08, 1.17)	2069 (1.02%)	2052 (1.01%)	0.99	0.93 to, 1.05

	Total events, N (cases/1000 person-months; 95% CI)	Safe group (N = 230,324), (cases/1000 person-months; 95% CI)^d^	Unsafe group (N = 234,002), (cases/1000 person-months; 95% CI)^d^	Safe group (N = 202,931), N (%)^d^	Unsafe group (N = 202,931), N (%)^d^	RR (mod Poisson)	
						Estimate	Estimate
**Risk ratios for three types of non-traumatic injuries in children aged 4 to 12 months (secondary outcome)**^**a**^							
Foreign body	7561 (1.81; 1.77,1.85)	3695 (1.79; 1.73, 1.85)	3866 (1.84; 1.78, 1.90)	3273 (1.61%)	3364 (1.66%)	1.02	0.98 to 1.07
Burn and frostbite	17,196 (4.11; 4.05,4.18)	**7991 (3.89; 3.80, 3.98)**^**†**^	**9205 (4.30; 4.20, 4.40)**^**†**^	**7109 (3.50%)**^**†**^	**7853 (3.87%)**^**†**^	**1.10**	**1.07 to 1.14**
Poisoning and toxic effects by drugs and other substances	1231 (0.29; 0.28,0.31)	575 (0.27; 0.25, 0.30)*	656 (0.30; 0.28, 0.33)*	500 (0.25%)	550 (0.27%)	1.10	0.97 to 1.24

Treatment^c^	Unmatched data (N = 464,326)			Matched data (N = 405,862)^b^			
	Safe group (N = 230,324), N (%)^d^	Unsafe group (N = 234,002), N (%)^d^		Safe group (N = 202,931), N (%)^d^	Unsafe group (N = 202,931), N (%)^d^	RR (mod Poisson)	
						Estimate	Estimate
**Risk ratios for four treatments received by children aged 4 to 12 months (additional outcome)**^**a**^							
Anesthesia	1626 (0.7%)*	1519 (0.6%)*		1,390 (0.7%)	1381 (0.7%)	0.99	0.92 to 1.07
Cast or splint	1016 (0.4%)	1079 (0.5%)		889 (0.4%)	939 (0.5%)	1.06	0.96 to 1.16
Transfusion	363 (0.2%)	320 (0.1%)		300 (0.1%)	292 (0.1%)	0.97	0.83 to 1.14
Ventilator supports	257 (0.1%)*	215 (0.1%)*		215 (0.1%)	199 (0.1%)	0.93	0.76 to 1.12
Total (at least one treatment)	2660 (1.2%)	2580 (1.1%)		2,296 (1.1%)	2307 (1.1%)	1.00	0.95 to 1.06

*N* number, *RR* relative risk, *CI* confidence interval.

^a^Unless otherwise specified, all treatments were assessed at the age of 4 to 12 months.

^b^Matched using inverse probability of exposure matching, based on propensity scores. The propensity score was estimated using multivariable logistic regression with covariates chosen a priori (Table S2).

^c^Treatments were based on NHIS codes (Supplementary Appendix 1).

^d^Values are reported as N (%) unless otherwise indicated.

*p < 0.05, ^†^p < 0.001.

Significant values are in bold.

### Secondary outcome: risk ratios for individual injuries

There were 53,321 infants (11.5%) with traumatic injuries, and the most common type was head and neck injury (36,205; 8.66 per 1000 person-months; 95% CI 8.57, 8.75). Further analysis of traumatic injuries showed that the risk ratios (RRs) were significantly greater in the “unsafe” group for head and neck injury (RR: 1.06; 95% CI 1.04, 1.09), trunk and abdominopelvic injury (RR: 1.12; 95% CI 1.04, 1.21), and upper extremity injury (RR: 1.04; 95% CI: 1.00, 1.07) in matched data.

We also examined the RRs for non-traumatic injuries from a foreign body, burn and frostbite, and poisoning in children who were 4 to 12 months-old. Among all 464,326 infants in the unmatched groups, there were 25,988 non-traumatic injures (5.6%). The most common non-traumatic injury was burn and frostbite, and this number was lower in the safe group (7991; 3.89 per 1000 person-months; 95% CI 3.80, 3.98) than in the unsafe group (9205; 4.30 per 1000 person-months; 95% CI 4.20, 4.40; p < 0.001) before matching. Poisoning and toxic injuries were statistically more common in the unsafe group before matching, but not after matching (Table 2).

### Additional outcome: risk ratios for treatments

We next examined the RRs for receipt of different treatments for injuries in infants who were 4 to 12 months-old. After matching, 2296 children in the safe group and 2307 infants in the unsafe group received at least 1 of the 4 analyzed treatments. Before matching, anesthesia and ventilator support were more common in the safe group, but this difference was not significant after matching (Table 2).

### Subgroup analysis

We further analyzed the RR for injury in infants who were 4 to 12 months-old according to specific items in the questionnaire (Table 3). The infants of parents who said they always used car seats were less likely to have wounds or fractures (RR: 0.96; 95% CI 0.95, 0.98). Also, the infants of parents who said they did not use a baby walker were less likely to have a wound or fracture (RR: 0.97; 95% CI 0.95, 0.98) and a foreign body injury (RR: 89; 95% CI 0.85, 0.94).

**Table 3:** Risk ratios for injury in children aged 4 to 12 months according to five questionnaire items (sensitivity analysis).

Childhood injuries^a^	Unmatched data (N = 464,326)		Matched data (N = 405,862)^c^		
	Yes, N (%)^c^, (N = 237,080)	No, N (%)^c^, (N = 207,444)	Yes, N (%)^c^, (N = 207,862)	No, N (%)^c^, (N = 180,753)	RR, (estimate, Wald 95% CI)
**Question 1: Do you always use a car seat when your child rides in a car? (yes: safe, no: unsafe)**					
Total injury (trauma or non-trauma)	40,858 (17.23%)*	34,989 (16.87%)*	35,822 (17.23%)*	30,437 (16.84%)*	0.98 (0.96 to 0.99)*
Wound/fracture	**30,038 (12.67%)**^**†**^	**25,262 (12.18%)**^**†**^	**26,286 (12.65%)**^**†**^	**22,055 (12.20%)**^**†**^	**0.96 (0.95 to 0.98)**^**†**^
Foreign body	3853 (1.63%)	3387 (1.63%)	3399 (1.64%)	2959 (1.64%)	1.00 (0.95 to 1.05)
Burn/frostbite	8606 (3.63%)*	7772 (3.75%)*	7573 (3.64%)	6692 (3.70%)	1.02 (0.98 to 1.05)
Poisoning	26 (0.01%)	21 (0.01%)	22 (0.01%)	18 (0.01%)	0.94 (0.50 to 1.75)

Childhood injuries^a^	Unmatched data (N = 444,524)		Matched data (N = 400,188)^b^		
	No, N (%)^c,^ (N = 240,829)	Yes, N (%)^c^, (N = 223,497)	No, N (%)^c^, (N = 211,707)	Yes, N (%)^c^, (N = 194,155)	RR, (estimate, Wald 95% CI)
**Question 2: Do you use a baby walker? (no: safe, yes: unsafe)**					
Total injury (trauma or non-trauma)	41,288 (17.14%)	38,021 (17.01%)	36,268 (17.13%)	32,958 (16.98%)	0.99 (0.98 to 1.01)
Wound/fracture	**30,448 (12.64%)**^**†**^	**27,332 (12.23%)**^**†**^	**26,732 (12.63%)**^**†**^	**23,726 (12.22%)**^**†**^	**0.97 (0.95 to 0.98)**^**†**^
Foreign body	**4117 (1.71%)**^**†**^	**3444 (1.54%)**^**†**^	**3649 (1.72%)**^**†**^	**2988 (1.54%)**^**†**^	**0.89 (0.85 to 0.94)**^**†**^
Burn/frostbite	**8397 (3.49%)**^**†**^	**8796 (3.94%)**^**†**^	**7365 (3.48%)**^**†**^	**7594 (3.91%)**^**†**^	**1.12 (1.09 to 1.16)**^**†**^
Poisoning	23 (0.01%)	26 (0.01%)	20 (0.01%)	22 (0.01%)	1.20 (0.65 to 2.20)

Childhood injuries^a^	Unmatched data (N = 464,326)		Matched data (N = 405,862)^b^		
	No, N (%)^c^, (N = 281,540)	Yes, N (%)^c^, (N = 182,786)	No, N (%)^c^, (N = 246,785)	Yes, N (%)^c^, (N = 159,077)	RR, (estimate, Wald 95% CI)
**Question 3: Have you ever left your child alone on the bed or couch for a while? (no: safe, yes: unsafe)**					
Total injury (trauma or non-trauma)	**45,982 (16.33%)**^**†**^	**33,327 (18.23%)**^**†**^	**40,283 (16.32%)**^**†**^	**28,943 (18.19%)**^**†**^	**1.11 (1.10 to 1.13)**^**†**^
Wound/fracture	**33,096 (11.76%)**^**†**^	**24,684 (13.50%)**^**†**^	**28,931 (11.72%)**	**21,527 (13.53%)**^**†**^	**1.15 (1.14 to 1.17)**^**†**^
Foreign body	4470 (1.59%)*	3091 (1.69%)*	3938 (1.60%)*	2699 (1.70%)*	1.16 (1.01 to 1.12) *
Burn/frostbite	**10,197 (3.62%)**^**†**^	**6996 (3.83%)**^**†**^	8989 (3.64%)	5970 (3.75%)	1.03 (1.00 to 1.06)
Poisoning	26 (0.01%)	23 (0.01%)	23 (0.01%)	19 (0.01%)	1.28 (0.70 to 2.35)

Childhood injuries^a^	Unmatched data (N = 464,326)		Matched data (N = 405,862)^b^		
	Yes, N (%)^c^, (N = 416,810)	No, N (%)^c^, (N = 47,516)	Yes, N (%)^c^, (N = 364,172)	No, N (%)^c^, (N = 41,690)	RR, (estimate, Wald 95% CI)
**Question 4: Do you keep an eye on your child all the time, including during playing and sleeping? (yes: safe, no: unsafe)**					
Total injury (trauma or non-trauma)	70,916 (17.01%)*	8393 (17.66%)*	61,883 (16.97%)*	7343 (17.61%)*	1.04 (1.01 to 1.06)*
Wound/fracture	51,645 (12.39%)*	6135 (12.91%)*	45,073 (12.36%)*	5385 (12.92%)*	1.04 (1.02 to 1.07)*
Foreign body	**6694 (1.61%)**^**†**^	**867 (1.82%)**^**†**^	5876 (1.61%)*	761 (1.83%)*	1.13 (1.05 to 1.22)*
Burn/frostbite	15,421 (3.70%)	1772 (3.73%)	13,433 (3.68%)	1526 (3.66%)	0.99 (0.94 to 1.05)
Poisoning	45 (0.01%)	4 (0.01%)	38 (0.01%)	4 (0.01%)	0.92 (0.33 to 2.58)

Childhood injuries^a^	Unmatched data (N = 464,326)		Matched data (N = 405,862)^b^		
	No, N (%)^c^, (N = 416,760)	Yes, N (%)^c^, (N = 47,566)	No, N (%)^c^, (N=364,540)	Yes, N (%)^c^, (N = 41,322)	RR, (estimate, Wald 95% CI)
**Question 5: Have you ever held a hot drink when carrying your child? (no: safe, yes: unsafe)**					
Total injury (trauma or non-trauma)	**70,422 (16.90%)**^**†**^	**8887 (18.68%)**^**†**^	**61,562 (16.89%)**^**†**^	**7664 (18.55%)**^**†**^	**1.10 (1.07 to 1.12)**^**†**^
Wound/fracture	**51,425 (12.34%)**^**†**^	**6355 (13.36%)**^**†**^	**44,962 (12.33%)**^**†**^	**5496 (13.30%)**^**†**^	**1.08 (1.05 to 1.11)**^**†**^
Foreign body	**6686 (1.60%)**^**†**^	**875 (1.84%)**^**†**^	**5868 (1.61%)**^**†**^	**769 (1.86%)**^**†**^	**1.16 (1.07 to 1.25)**^**†**^
Burn/frostbite	**15,082 (3.62%)**^**†**^	**2111 (4.44%)**^**†**^	**13,156 (3.61%)**^**†**^	**1803 (4.36%)**^**†**^	**1.21 (1.15 to 1.27)**^**†**^
Poisoning	44 (0.01%)	5 (0.01%)	37 (0.01%)	5 (0.01%)	1.19 (0.47 to 3.03)

*N* number, *CI* confidence interval.

Unless otherwise specified, all diagnoses of childhood were assessed at eh age of 4 to 12 months (after the first, and before the second round of the NHSPIC).

^a^Diagnosis of childhood injuries was based on ICD-10 codes (Supplementary Appendix 2).

^b^Matched using inverse probability of exposure matching, based on propensity scores. The propensity score was estimated using multivariable logistic regression with covariates chosen a priori (Table S2).

^c^Values are reported as N (%) unless otherwise indicated.

*p < 0.05, ^†^p < 0.001.

Significant values are in bold.

The infants of parents who said they left their children alone were more likely have a wound or fracture (RR: 1.15; 95% CI 1.14, 1.17) and a foreign body injury (RR: 1.06; 95% CI 1.01, 1.12). The infants of parents who said they did not always keep an eye on their infants were more likely to experience a wound or fracture (RR: 1.04; 95% CI 1.02, 1.07) and a foreign body injury (RR: 1.13; 95% CI 1.05, 1.22). The infants of parents who answered that they held hot drinks when carrying their infants had more wounds and fractures (RR: 1.08; 95% CI: 1.05, 1.11), foreign body injuries (RR: 1.16; 95% CI 1.07, 1.25), and burns or frostbite (RR: 1.21; 95% CI 1.15, 1.27).

In addition, we examined the RR of childhood injuries according to each ICD code (S00 to S99 and T15 to T78; Supplementary Table S4). Head injuries were the most common (31,259, 15.4%), and matched analysis indicated these were more common in the unsafe group (p < 0.001). Matched analysis also indicated that injuries of the thorax, shoulder and upper arm, elbow and forearm, and burns were more common in the unsafe group (all p < 0.05).

### Sensitivity analysis

Finally, we performed a sensitivity analysis by adding the missing data, as described in the Methods. In the unmatched data, 75,624 of 444,052 infants (17.0%) had injuries. There were significant differences between safe and unsafe groups in the diagnosis of total injuries in the matched data (16.6 *vs.* 17.4%, p < 0.001) and in the unmatched data (16.6 *vs.* 17.4%, p < 0.001). The results of this analysis were consistent with those of the main cohort (Supplementary Table S5).

## Discussion

Developed countries have growing concerns about issues related to the safety and injury of young children. The present study showed that careful parental supervision during infancy led to fewer traumatic injuries of the abdomen and extremities, and fewer burn-related injuries. Notably, infants whose parents did not leave them alone and always kept their eyes on them had fewer wounds and fractures and foreign body injuries, and infants whose parents did not hold a hot drink when carrying them had fewer burn injuries. These findings emphasize the importance of careful parental supervision of infants when they are 4 to 6 months-old because this may be associated with decreased prevalence of safety accidents in some part of injury mechanism when they are 6 to 12 months-old. Our results are especially meaningful because we analyzed the effects of preventive measures using a nationwide health care program with population-based data. Thus, developers of health policies and primary care physicians should consider these findings in their future efforts to prevent injuries during childhood.

Identification of what parents should do to prevent injuries, how well different injury prevention strategies work, and how best to encourage parents to adopt effective strategies are essential to decrease the risk of childhood injury^15^. Our study used nationwide population-based data to assess the preventive effects of five specific parental behaviors on infant injuries.

Previous research showed that parental supervision improved the child safety and reduced injuries in many ways. An epidemiological study in Bangladesh reported that children under 5 years-old who died due to unintentional injuries had a 3.3-times increased likelihood of being unsupervised relative to other children^16^. However, this study only examined the injury outcome of death, and did not consider the causes of injuries (drowning, traffic injuries, suffocation, cuts, poisoning, and animal bite). A population-based birth cohort study of young children in Japan reported that a high degree of paternal involvement in childcare at 6 months of age led to fewer accidental injuries at age 18 months, and that children whose fathers took them for walks had fewer accidental injuries^17^. A meta-analysis found that familial interventions significantly lowered the risk of childhood injury from birth to age 8 years as measured by self-reports, and suggested that familial interventions prevented injuries and improved the safety of children at the home^11^.

Our study utilized scores from a safety-related questionnaire, multiple statistical methods, and large amount of medical information (ICD-10 codes from medical insurance data) to achieve a highly reliable evaluation of safety-related parental supervision practices. In addition, we focused on infants up to the age of 12 months to assess the preventive effects of parental supervision on childhood injuries. Infants are generally not responsible for their own injuries, and the parents have nearly all the responsibility to keep them safe. Thus, parents should have an increased awareness that they need to provide a safe environment and appropriate supervision of their infants to prevent injuries. We also considered the practical effects of specific parental behaviors by separate analysis of each of the 5 safety-related supervision activities. This additional analysis was conducted because the different questions evaluated different specific behaviors of the parents. We found that infant injuries were reduced when parents did not leave their children alone, always kept an eye on their children, and did not handle a hot drink while carrying their infants. This indicates that parental practices, attitudes, and behaviors related to safety significantly affected injuries in infant from the age of 4 to 12 months.

Parents can play an important role in reducing the risk of injury in their children by providing more close supervision^7^. Several interventions by the parents can significantly reduce the risk of childhood injuries and improve safety in the home^11^. For example, parents can play a valuable role by preventing a child’s hazardous activities, and the extent of parental supervision appears to be related to a child’s risk for injury^7,18^. Moreover, participation of the father in childcare and supervision can prevent injuries in young children, possibly because it reduces maternal stress^17^. Educating parents about how to prevent childhood injuries will increase their awareness of potential injuries so they can increase the use of safe products and performance of safe activities by their children. This education can be implemented in a number of settings, including primary care offices, community-based organizations, schools, and emergency departments^9^. To develop and implement effective strategies for injury prevention in children, it is necessary to understand what parents can do to prevent injuries, the efficacy of different strategies, and how to encourage parents to adopt the most effective strategies^15^. Our findings emphasize that caregivers must provide vigilant supervision to increase the safety and reduce the risk of injuries in children.

It is widely believed that parental knowledge of the importance of safe interventions, such as using a car seat, are essential for keeping children safe. In particular, 25 to 46% of children who died in motor vehicle accidents were unrestrained, and the correct installation and utilization of a cart seat reduces the risk of fatal injury by 71% in infants and 54% in toddlers (age 1–3 years)^19–22^. However, we found that use of a car seat was not associated with the prevalence of wounds or fracture injuries in infants. Nonetheless, because so many other studies have reported the importance of car seats, the need for their use should not be underestimated.

Traditional parental guidance and previous research recommended against using baby walkers for young children because they increase the risk of physical injuries^23^. In contrast, we found that prohibiting a baby walker did not decrease the prevalence of physical injuries in young children. It is possible that the more recently produced baby walkers are safer than the older ones, or that parents are more aware of the risk of baby walkers, due to reports in the media and elsewhere, and therefore use more caution.

To the best of our knowledge, the present study is the first to examine the effect of parental supervision on the risk of infant injuries and treatments for injuries that analyzed a large population-based cohort of more than 400,000 infants. The outcomes were not limited to a single result, such as death. Instead we considered physical trauma to different regions of the body, non-traumatic injuries by a foreign body, burn and frostbite, and poisoning, and injuries necessitating anesthesia, a cast or splint, transfusion, and ventilator support using the extensive objective data of the NHIS of Korea. Because there are age-related differences in childhood injuries, we focused on infants, a time when injury prevention measures by parents may be most important. We anticipate that our findings of the beneficial effects of parental supervision during infancy might provide a cornerstone for developing parental supervision practices for older children.

This study has several limitations. First, we used a relatively simple questionnaire. Ideally, the questionnaire should have more carefully considered the developmental stage of the children. The presence of unanticipated communication barriers between the investigators and parents could have led to inaccurate responses. Our questionnaire asked the parents whether they left their infants alone while on a bed or the couch, and whether they kept an eye on the infant at all times, even while sleeping. However, expecting this level of parental supervision may be unrealistic, and an answer of “no” to this question does not necessarily mean the infant has an increased risk of injury. In addition, we could not analyze or compare the variables in the groups who did not respond to the questionnaires. Second, we did not consider psychological factors or mental illnesses in the parents, such as depression, substance use disorder, and anxiety, and these have known effects on the risk of injury in children. A study in Sweden reported that exposure to a mentally ill parent during infancy was responsible for an approximately 30% increased risk for any type of injury in infants, more than any other factor these researchers analyzed^24^. Unfortunately, we could not assess psychological factors or mental illness in the parents because the NHSPIC data did not have this information.

In conclusion, our study showed that greater parental supervision was associated with reduced prevalences of injuries and reduced need for injury treatments in infants. Implementation of an education program that encourages parents to provide more supervision to their children may help to prevent childhood injuries and reduce the social and financial costs at the local and nation-wide level. Further studies of other measures that can be used to prevent injuries during childhood by using a health screening program for children of other ages are also needed to improve the health and welfare of Korean children.

## Supplementary Information

Supplementary Information.

## Data Availability

The datasets used during the current study available from the corresponding author on reasonable request.
