# Supplementary material for: Effect of parental supervision of infants at age 4 to 6 months on injuries at age 4 to 12 months
Source: Sci Rep. 2022 Jun 17;12:10252. doi: 10.1038/s41598-022-14321-8 (PMC9205875; doi:10.1038/s41598-022-14321-8)
Supplement: Supplementary file 1 — Supplementary Information. [file 41598_2022_14321_MOESM1_ESM.docx]

**Supplementary Table S1.** Safety-related questionnaire administered to parents when their children were 4 to 6 months-old (top) and results from this questionnaire (bottom).

| **Question** | | Safe Group | Unsafe Group | Total |
| --- | --- | --- | --- | --- |
| Q1. | Do you always use a car seat when your child rides in a car? (yes: 0, no: 1) | Yes: 207,712 (46.7%) | No: 237,411 (53.3%) | 445,123 |
| Q2. | Do you use a baby walker? (no: 0, yes: 1) | No: 241,503 (51.9%) | Yes: 224,172 (48.1%) | 465,675 |
| Q3. | Have you ever left your child alone on the bed or couch for a while? (no: 0, yes: 1) | No: 283,349 (60.7%) | Yes: 183,338 (39.3%) | 466,687 |
| Q4. | Do you keep an eye on your child all the time, including when playing and sleeping? (yes: 0, no: 1) | Yes: 47,669 (10.2%) | No: 418,088 (89.8%) | 465,757 |
| Q5. | Have you ever held a hot drink when you carrying your child? (no: 0, yes: 1) | No: 418,001 (89.7%) | Yes: 47,754 (10.3%) | 465,755 |

| Total questionnaire score | Safe or unsafe group | Parents of study subjects who  answered all or some questions, N (%) | Parents of study subjects who  answered all questions, N (%) |
| --- | --- | --- | --- |
| 0 | Safe | 78,549 (16.9%) | 78,549 (16.9%) |
| 1 | Safe | 152,099 (32.6%) | 151,775 (32.7%) |
| 2 | Unsafe | 147,200 (31.6%) | 146,513 (31.6%) |
| 3 | Unsafe | 68,651 (14.7%) | 68,239 (14.7%) |
| 4 | Unsafe | 17,309 (3.7%) | 17,157 (3.7%) |
| 5 | Unsafe | 2,120 (0.5%) | 2,093 (0.5%) |
| Total |  | 465,928 | 464,326 |

**Supplementary Table S2.** Covariates included in the 1:1 propensity score model.

| Category | Covariates |
| --- | --- |
| Demographic characteristics | Sex; Birth residence (Seoul, Metropolitan, City, Rural); Income quintile. |
| Clinical characteristics (perinatal period)^a^ | Prematurity; Birth weight; Conditions that originated during the perinatal period (maternal factors, disorders related to length of gestation and fetal size, birth traumas, respiratory and cardiovascular disorders specific to the perinatal period, infections specific to the perinatal period, hemorrhagic and hematological disorders of the fetus and newborn, transitory endocrine and metabolic disorders, digestive system disorders of the fetus and newborn, conditions involving the integument and temperature regulation, congenital malformations, deformations and other disorders originating in the perinatal period, and chromosomal abnormalities).^*^ |
| Diagnosis of injury  (before age 4 months)^b^ | Injuries to the head; Injuries to the neck; Injuries to the thorax; Injuries to the abdomen, lower back, lumbar spine and pelvis; Injuries to the shoulder and upper arm; Injuries to the elbow and forearm; Injuries to the wrist and hand; Injuries to the hip and thigh; Injuries to the lower knee and lower leg; Injuries to the ankle and foot; Injuries involving multiple body regions; Injuries to an unspecified part of the trunk, limb or body region; Injury from a foreign body; Burn; Frostbite; Poisoning by drugs, medicaments and biological substances; Toxic effects of substances chiefly nonmedicinal as to source; Unspecified effects of external causes. |
| Treatments  (before age 4 months)^c^ | Anesthesia; Cast or splint; Transfusion; Ventilator support. |

^a^ Conditions that originated during the perinatal period were defined by ICD-10 codes (Appendix 1).

^b^ Childhood injuries were defined by ICD-10 codes (Appendix 2).

^c^ Treatments were defined by NHIS codes (Appendix 3).

**Supplementary Table S3.** Injuries and treatments when children were less than 4 months-old.^a^

|  | Unmatched data (N=464,326) | | | |  | Matched data (N=405,862)^b^ | | |  |
| --- | --- | --- | --- | --- | --- | --- | --- | --- | --- |
|  | Safe Group  N (%)^c^, (N=230,324) | Unsafe Group  N (%)^c^, (N=234,002) | Standardized Difference, %^d^ | |  | Safe Group  N (%)^c^, (N=202,931) | Unsafe Group  N (%)^c^, (N=202,931) | Standardized Difference, % ^d^ | |
| **Diagnosis**^e^ |  |  | |  |  |  |  |  | |
| Injuries to the head | 1,419 (0.6%) | 1,621 (0.7%) | | −0.930 |  | 1,283 (0.6%) | 1,390 (0.7%) | −0.657 | |
| Injuries to the neck | 47 (0.0%) | 54 (0.0%) | | −0.160 |  | 41 (0.0%) | 48 (0.0%) | −0.2370 | |
| Injuries to the thorax | 50 (0.0%) | 60 (0.0%) | | −0.354 |  | 46 (0.0%) | 46 (0.0%) | 0 | |
| Injuries to the abdomen, lower back, lumbar spine, and pelvis | 114 (0.0%) | 133 (0.1%) | | −0.349 |  | 102 (0.1%) | 116 (0.1%) | −0.298 | |
| Injuries to the shoulder and upper arm | 487 (0.2%) | 437 (0.2%) | | 0.533 |  | 360 (0.2%) | 408 (0.2%) | −0.530 | |
| Injuries to the elbow and forearm | 188 (0.1%) | 219 (0.1%) | | −0.405 |  | 172 (0.1%) | 176 (0.1%) | −0.067 | |
| Injuries to the wrist and hand | 314 (0.1%) | 332 (0.1%) | | −0.287 |  | 273 (0.1%) | 300 (0.1%) | −0.359 | |
| Injuries to the hip and thigh | 147 (0.1%) | 117 (0.0%) | | 0.573 |  | 106 (0.1%) | 110 (0.1%) | −0.083 | |
| Injuries to the lower knee and lower leg | 77 (0.0%) | 70 (0.0%) | | 0.232 |  | 60 (0.0%) | 67 (0.0%) | −0.192 | |
| Injuries to the ankle and foot | 142 (0.1%) | 171 (0.1%) | | −0.409 |  | 129 (0.1%) | 143 (0.1%) | −0.267 | |
| Injuries involving multiple body regions | 128 (0.1%) | 139 (0.1%) | | −0.257 |  | 116 (0.1%) | 127 (0.1%) | −0.225 | |
| Injuries to unspecified part of trunk, limb or body region | 344 (0.1%) | 300 (0.1%) | | 0.596 |  | 255 (0.1%) | 286 (0.1%) | −0.409 | |
| Foreign body injury | 695 (0.3%) | 656 (0.3%) | | 0.471 |  | 574 (0.3%) | 614 (0.3%) | −0.366 | |
| Burn | 205 (0.1%) | 242 (0.1%) | | −0.429 |  | 184 (0.1%) | 212 (0.1%) | −0.448 | |
| Frostbite | 1 (0.0%) | 0 (0.0%) | |  |  | 0 (0.0%) | 0 (0.0%) |  | |
| Poisoning by drugs and biological substances | 1 (0.0%) | 1 (0.0%) | | 0 |  | 1 (0.0%) | 1 (0.0%) | 0 | |
| Toxic effects of nonmedicinal substances | 32 (0.0%) | 24 (0.0%) | | 0.264 |  | 20 (0.0%) | 24 (0.0%) | −0.179 | |
| Unspecified effects of external causes | 205 (0.1%) | 221 (0.1%) | | −0.186 |  | 180 (0.1%) | 203 (0.1%) | −0.376 | |
| **Treatment**^f^ |  |  | |  |  |  |  |  | |
| Anesthesia | 1,533 (0.7%) | 1,387 (0.6%) | | 0.900 |  | 1,179 (0.6%) | 1,291 (0.6%) | −0.701 | |
| Cast of splint | 135 (0.1%) | 111 (0.0%) | | 0.412 |  | 93 (0.0%) | 104 (0.1%) | −0.236 | |
| Transfusion | 1,526 (0.7%) | 1,235 (0.5%) | | 1.708 |  | 1,091 (0.5%) | 1,166 (0.6%) | −0.481 | |
| Ventilator | 2,353 (1.0%) | 1,982 (0.8%) | | 1.743 |  | 1,724 (0.8%) | 1,876 (0.9%) | −0.781 | |

Abbreviations: N, number; ICD, International Classification of Diseases.

^a^ Unless otherwise specified, all diagnoses and treatments were assessed in children less than 4 months-old.

^b^ Matched using the inverse probability of exposure matching, based on propensity scores. The propensity score was estimated using multivariable logistic regression with covariates chosen *a priori* (eTable 1).

^c^ Values are reported as N (%) unless otherwise indicated.

^d^ The difference between groups divided by the pooled standard deviation; a value greater than 10% was considered meaningful.

^e^ Childhood injuries were identified by ICD-10 codes (Appendix 2)

^f^ Treatments of childhood injuries were identified by NHIS codes (Appendix 3).

**Supplementary Table S4.** Risk of different injuries in children from age 4 to 12 months.^a^

|  | Unmatched data (N=464,326) | | |  | Matched data (N=405,862)^b^ | | |  |
| --- | --- | --- | --- | --- | --- | --- | --- | --- |
|  | Safe group  (N=230,324),  N (%)^d^, | Unsafe group  (N=234,002),  N (%)^d^ | *P* value |  | Safe group  (N=202,931),  N (%)^d^ | Unsafe group  (N=202,931),  N (%)^d^ | *P* value | |
| **Childhood injury**^d^ |  |  |  |  |  |  |  | |
| Head | 17,250 (7.5%) | 18,535 (7.9%) | **<0.001†** |  | 15,145 (7.5%) | 16,114 (7.9%) | **<0.001†** | |
| Neck | 249 (0.1%) | 251 (0.1%) | 0.930 |  | 217 (0.1%) | 214 (0.1%) | 0.885 | |
| Thorax | 214 (0.1%) | 274 (0.1%) | **0.011^*^** |  | 188 (0.1%) | 231 (0.1%) | **0.036^*^** | |
| Abdomen, lower back, lumbar spine and pelvis | 316 (0.1%) | 338 (0.1%) | 0.510 |  | 280 (0.1%) | 292 (0.1%) | 0.616 | |
| Shoulder and upper arm | 923 (0.4%) | 1,053 (0.4%) | **0.010^*^** |  | 811 (0.4%) | 910 (0.4%) | **0.017^*^** | |
| Elbow and forearm | 3,584 (1.6%) | 3,947 (1.7%) | **<0.001†** |  | 3,153 (1.6%) | 3,413 (1.7%) | **0.001^*^** | |
| Wrist and hand | 4,419 (1.9%) | 4,481 (1.9%) | 0.928 |  | 3,889 (1.9%) | 3,894 (1.9%) | 0.954 | |
| Hip and thigh | 691 (0.3%) | 656 (0.3%) | 0.213 |  | 591 (0.3%) | 589 (0.3%) | 0.954 | |
| Lower knee and lower leg | 445 (0.2%) | 451 (0.2%) | 0.971 |  | 388 (0.2%) | 412 (0.2%) | 0.396 | |
| Ankle and foot | 1,282 (0.6%) | 1,278 (0.5%) | 0.630 |  | 1,127 (0.6%) | 1,091 (0.5%) | 0.443 | |
| From a foreign body | 3,695 (1.6%) | 3,866 (1.7%) | 0.198 |  | 3,273 (1.6%) | 3,364 (1.7%) | 0.260 | |
| Burn | 7,989 (3.5%) | 9,204 (3.9%) | **<0.001†** |  | 7,107 (3.5%) | 7,852 (3.9%) | **<0.001†** | |
| Frostbite | 3 (0.0%) | 2 (0.0%) | 0.642 |  | 2 (0.0%) | 2 (0.0%) | 1.000 | |
| Poisoning by drugs and biological substances | 19 (0.0%) | 30 (0.0%) | 0.130 |  | 16 (0.0%) | 26 (0.0%) | 0.123 | |
| Toxic effects of nonmedicinal substances | 442 (0.2%) | 489 (0.2%) | 0.194 |  | 387 (0.2%) | 405 (0.2%) | 0.522 | |
| Unspecified effects of external causes | 114 (0.0%) | 139 (0.1%) | 0.148 |  | 97 (0.0%) | 121 (0.1%) | 0.104 | |

Abbreviations, N, Number; ICD, International Classification of Diseases

^a^ Unless otherwise specified, all of diagnoses were assessed when the children were 4 to 12 months-old.

^b^ Matched using inverse probability of exposure matching based on propensity scores. The propensity score was estimated using multivariable logistic regression with covariates chosen *a priori* (eTable 1).

^c^ Values are reported as N (%) unless otherwise indicated.

^d^ The diagnosis of childhood injuries was from ICD-10 codes (Appendix 2).

(^*:^ *p* <.05, †: p<0.001)

**Supplementary Table 5.** Risk ratios for injuries and treatments in children aged 4 to 12 months (added missing data) (N=444,052)

| Diagnosis of total injury (trauma or non-trauma) in children aged 4 to 12 months. ^a^ | | | | | | | | |
| --- | --- | --- | --- | --- | --- | --- | --- | --- |
|  | Unmatched data (N=444,052) | | |  | Matched data (N=420,400)^b^ | | | |
|  | Total events, N (%) | Safe Group  (N=220,075), N (%)^d^ | Unsafe Group (N=223,977), N (%)^d^ |  | Safe Group (N=210,200),(%)^d^ | Unsafe Group (N=210,200),(%)^d^ | RR (mod Poisson) | |
|  |  |  |  |  |  |  | Estimate | Wald 95% CI |
| Total injury (trauma or non-trauma) | 75,624 (17.0%) | **36,456 (16.6%)†** | **39,168 (17.4%)†** |  | **34,890 (16.6%)†** | **36,663 (17.4%)†** | **1.05** | **1.04** to **1.07** |
| Risk ratios for traumatic injuries of different body legions in children aged 4 to 12 months. ^a^ | | | | | | | | |
|  | Unmatched data (N=464,326) | | |  | Matched data (N=405,862)^b^ | | | |
|  | Total events, N (%) | Safe Group  (N=220,075), N (%)^d^ | Unsafe Group (N=223,977), N (%)^d^ |  | Safe Group (N=210,200),(%)^d^ | Unsafe Group (N=210,200),(%)^d^ | RR (mod Poisson) | |
|  |  |  |  |  |  |  | Estimate | Wald 95% CI |
| Head and neck injury | 34,520 (7.8%) | **16,638 (7.6%)†** | **17,882 (8.0%)†** |  | **15,920 (7.6%)†** | **16,717 (8.0%)†** | **1.05** | **1.03** to **1.07** |
| Trunk and abdominopelvic injury | 2,926 (0.7%) | **1,365 (0.6%)*** | **1,561 (0.7%)*** |  | **1,304 (0.6%)*** | **1,479 (0.7%)*** | **1.13** | **1.05** to **1.22** |
| Upper extremity injury | 15,425 (3.5%) | **7,512 (3.4%)*** | **7,913 (3.5%)*** |  | 7,177 (3.4%) | 7,382 (3.5%) | 1.03 | 1.00 to 1.06 |
| Hip and lower extremity injury | 4,508 (1.0%) | 2,267 (1.0%) | 2,241 (1.0%) |  | 2,167 (1.0%) | 2,112 (1.0%) | 0.97 | 0.92 to 1.03 |
| Risk ratios for three types of non-traumatic injuries in children aged 4 to 12 months. ^a^ | | | | | | | | |
|  | Total events, N (%) | Safe Group  (N=220,075), N (%)^d^ | Unsafe Group (N=223,977), N (%)^d^ |  | Safe Group (N=210,200),(%)^d^ | Unsafe Group (N=210,200),(%)^d^ | RR (mod Poisson) | |
|  |  |  |  |  |  |  | Estimate | Estimate |
| Foreign body | 7,207 (1.6%) | 3,534 (1.6%) | 3,673 (1.6%) |  | 3,394 (1.6%) | 3,452 (1.6%) | 1.01 | 0.97 to 1.07 |
| Burn and frostbite | 16,392 (3.7%) | **7,613 (3.5%)†** | **8,779 (3.9%)†** |  | **7,295 (3.5%)†** | **8,201 (3.9%)†** | 1.12 | 1.09 to 1.16 |
| Poisoning and toxic effects by drugs and other substances | 1,164 (0.3%) | 546 (0.2%) | 618 (0.3%) |  | **518 (0.2%)*** | **583 (0.3%)*** | 1.12 | 1.00 to 1.27 |

Abbreviations: N, number; RR relative risk; CI, confidence interval.

^a^ Unless otherwise specified, all treatments were assessed at the age of 4 to 12 months .

^b^ Matched using inverse probability of exposure matching, based on propensity scores. The propensity score was estimated using multivariable logistic regression with covariates chosen *a priori* (eTable 2).

^c^ Treatments were based on NHIS codes (Appendix 1).

^d^ Values are reported as N (%) unless otherwise indicated.

*: *p* < 0.05, †: p < 0.001

**Appendix 1.** NHIS codes of treatments for childhood injuries.

| Treatment | NHIS code |
| --- | --- |
| Anesthesia | L1211 |
| Cast or splint | T6010-T6163 |
| Transfusion | X2021-X2022 |
| Ventilator support | M5850-M5858, M5860 |

**Appendix 2.** ICD-10 diagnostic codes of childhood injuries.

| Diagnosis | ICD-10 code |
| --- | --- |
| Injuries to the head | S00-S09 |
| Injuries to the neck | S10-S19 |
| Injuries to the thorax | S20-29 |
| Injuries to the abdomen, lower back, lumbar spine and pelvis | S30-S39 |
| Injuries to the shoulder and upper arm | S40-S49 |
| Injuries to the elbow and forearm | S50-S59 |
| Injuries to the wrist and hand | S60-S69 |
| Injuries to the hip and thigh | S70-S79 |
| Injuries to the lower knee and lower leg | S80-S89 |
| Injuries to the ankle and foot | S90-S99 |
| Foreign body in body | T15-T19 |
| Burn | T20-T32 |
| Frostbite | T33-T35 |
| Poisoning by drugs, medicaments and biological substances | T36-T49 |
| Toxic effects of substances chiefly nonmedicinal as to source | T50-T65 |
| Unspecified effects of external causes | T66-T78 |

**Appendix 3.** ICD-10 diagnostic codes of conditions that originated during the perinatal period.

| Condition | ICD-10 code |
| --- | --- |
| Maternal factors | P00-P049 |
| Disorders related to length of gestation and fetus | P05-P089 |
| Birth trauma | P10-P159 |
| Respiratory and cardiovascular disorder in perinatal period | P20-P299 |
| Infections in the perinatal period | P35-P399 |
| Hemorrhagic and hematological disorders in perinatal period | P50-P619 |
| Endocrine and metabolic disorders in perinatal period | P70-P749 |
| Digestive disorders in perinatal period | P75-P789 |
| Integument and temperature regulation related problems | P80-P849 |
| Congenital malformations or deformations | P90-P969 |
| Chromosomal abnormality | Q00-Q999 |
